# Low Power Wireless Smoke Alarm System in Home Fires

**DOI:** 10.3390/s150820717

**Published:** 2015-08-21

**Authors:** Juan Aponte Luis, Juan Antonio Gómez Galán, Javier Alcina Espigado

**Affiliations:** 1 OnTech Security LLC, C/Hispano Aviación, 7-9, 41300 La Rinconada, Sevilla, Spain; E-Mails: juan.aponte@ontech.es (J.A.L.); javier.alcina@ontech.es (J.A.E.); 2 Department Electronic Engineering, Computer Systems, and Automatics, University of Huelva, Ctra Huelva, La Rábida, s/n, 21819 Huelva, Spain

**Keywords:** fire detector, smoke sensor, wireless sensor network, low power consumption

## Abstract

A novel sensing device for fire detection in domestic environments is presented. The fire detector uses a combination of several sensors that not only detect smoke, but discriminate between different types of smoke. This feature avoids false alarms and warns of different situations. Power consumption is optimized both in terms of hardware and software, providing a high degree of autonomy of almost five years. Data gathered from the device are transmitted through a wireless communication to a base station. The low cost and compact design provides wide application prospects.

## 1. Introduction

Home fire detection is a matter of great concern, and thus many efforts are devoted in most developed countries to the design of automatic detection systems [1,2]. A fire alarm system should reliably and in a timely way notify building occupants about the presence of fire indicators, such as smoke or high temperatures. A fire detector is usually implemented as a smoke sensor [3,4,5] due to its early fire detection capability, fast response time and relatively low cost. Other options for the fire detection are based on gas sensors [6,7] or temperature sensors [8]. Fire detectors that use a single sensor, generally a smoke sensor, present high false-alarm rates due to temperature changes [9]. The smoke sensor is based on infrared (IR) light refraction due to the presence of smoke into a small chamber. Both IR LED and photodiode are temperature dependent and thus the sensitivity of the smoke sensor also depends on the ambient temperature, although this effect is cancelled in high performance devices [10]. Therefore, a fire detector that combines several types of sensors provides a more efficient fire alarm system [11].

Conventional approaches are based on wired systems, such as CAN bus, due to the high safety in critical applications. Although bus network have greatly improved in expansibility and maintenance, wireless systems have become more attractive in recent years offering a low-cost solution and spatial flexibility. A wireless sensor network requires sensor nodes small in size to facilitate the deployment and limited power consumption due to their battery-powered operation [12,13,14,15,16]. A wireless fire system must guarantee the functionality and the safety of the RF communications, avoiding false alarm notifications. Moreover, the system must notify the failure of components, physical damage or attempted sabotage, facilitating maintenance and thus reducing unnecessary costs.

## 2. Sensing Device Description

The developed sensing device allows deploying a wireless network where the data gathered are sent to a base station for further processing. The base station serves as the gateway between sensor nodes and users. A mobile application has also been developed to notify the user in real time about a fire alarm. The wireless sensor network collects sensing data of a home fire, analyzes the sensing data and accurately triggers a fire alarm. The design meets the required small size and low power consumption demands for a wireless node. The deployment of a high number of sensing nodes also needs a low cost solution. The system performs different parameter measurements for early detection of home fires. The node includes analog sensors to measure smoke, carbon monoxide (CO) and temperature.

**Figure 1:**
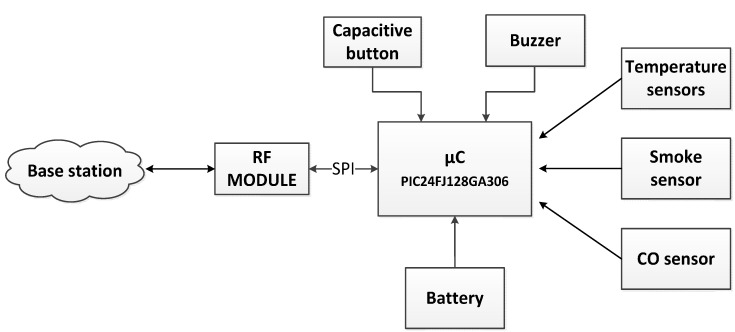
Building blocks of the sensing node.

Figure 1 shows the building blocks of the developed device. It consists of a microcontroller, a short-range radio transceiver, a battery, a CO sensor, a smoke sensor, temperature sensors, a capacitive touch button, a LED of red color and a buzzer. The entire system is protected by a specific protection box.

The design of the fire detection device is distributed on two printed circuit boards. The simplest of them includes the circuitry to handle and control a touch button and a buzzer, while the main board includes all required fire detection circuits, light indicator, data processing and wireless data transmission.

The main board is the most important part of the sensing device since it gathers all the smoke, gas and temperature measurements and sends them to the control central as alarms. The appropriate energy handling requires low power electronics for sensors, microcontroller, conditioning interfaces and transceiver. The microcontroller chosen was the PIC24FJ128GA306 from Microchip (Chandler, AZ, USA). The radio module exchanges data with a base station node via wireless at 868 MHz, following the standard IEEE 802.15.4. The base station node acts as a gateway between the sensor network and the user (or offices if the system is used for industrial applications). The power supply consists of one AA lithium CR123-3V 1600 mAh battery. A great autonomy of the sensing device has been guaranteed with a careful hardware since the design has been made choosing elements that ensure proper functionality with low power consumption. In addition, the software also incorporates save energy modes. The battery lasts around 5 years.

*Radio module*: The radio module is based on the MRF89XA transceiver from Microchip, one of the lower power consumption devices available. The MCP9700A temperature sensor is a low-cost, low power analog sensor which does not require an additional signal-conditioning circuit. The voltage output is directly connected to the ADC input of the microcontroller. This sensor is suitable for applications that require measurement of a relative change of temperature.

**Figure 2:**
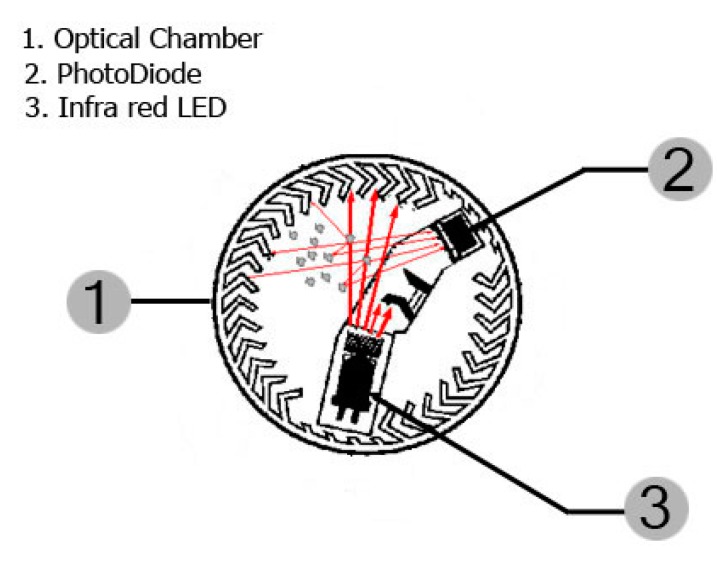
Chamber of the smoke sensor.

*Smoke sensor*: This sensor is responsible for detecting smoke through opto-electronic techniques and operates on the light scattering principle. It uses a beam of light emitted by a SFH4551 light emitting diode (LED) from Osram (Regensburg, Baviera, Germany) driven in a dark chamber that prevents the receiver (a SFH2500 photodiode, from Osram) from detecting the passage of light due to absorption of the light by the black material. In this chamber, when smoke particles enter the light path, light strikes the particles and is reflected onto the photosensitive device alerting the microcontroller. Figure 2 shows the chamber geometry.

**Figure 3:**
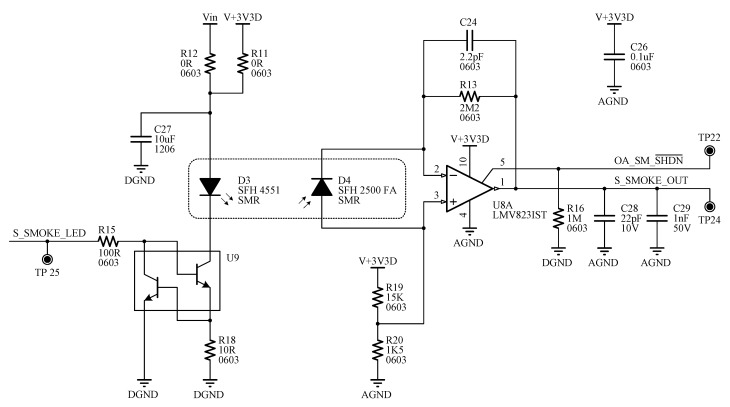
Signal-conditioning interface of the smoke sensor.

Figure 3 shows the circuit scheme of the smoke sensor. This topology is very attractive because it requires fewer components than the conventional way [17,18]. The photodiode operates with zero bias resulting in a most precise linear operation and lower noise. Moreover, this configuration is more suitable for precision applications at the expense of switching speed. The current of the photodiode is converted into a usable voltage using only one op amp as a current to voltage converter [19]. The feedback resistor is set to meet the required sensitivity range. A compensation capacitor is also placed in the feedback loop.

**Figure 4:**
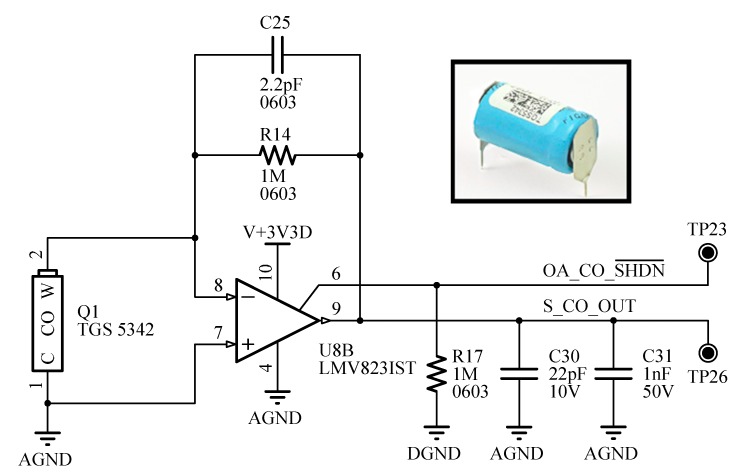
Scheme of the CO sensor.

*CO sensor*: This sensor measures the level of carbon monoxide typically produced in the combustion of materials in a home fire. The TGS5342 sensor from Figaro (Minoo, Osaka, Japan) has been chosen because it features a compact size, long life, good long term stability and high accuracy. It can detect concentrations as high as 1% CO in a wide range of temperatures. This sensor is critical to differentiate smoke from such water vapor or smoke produced from burning wood. Figure 4 shows the basic measuring circuit of the CO sensor based on a basic transresistance amplifier.

### 2.1. Sensor Data Acquisition

The microcontroller governs the wireless sensing device and controls the data acquisition from the specified sensors, the signal processing, data management and communications. The output signal of the analog sensors is converted to a binary value using the analog to digital converter of the microcontroller.

**Figure 5:**
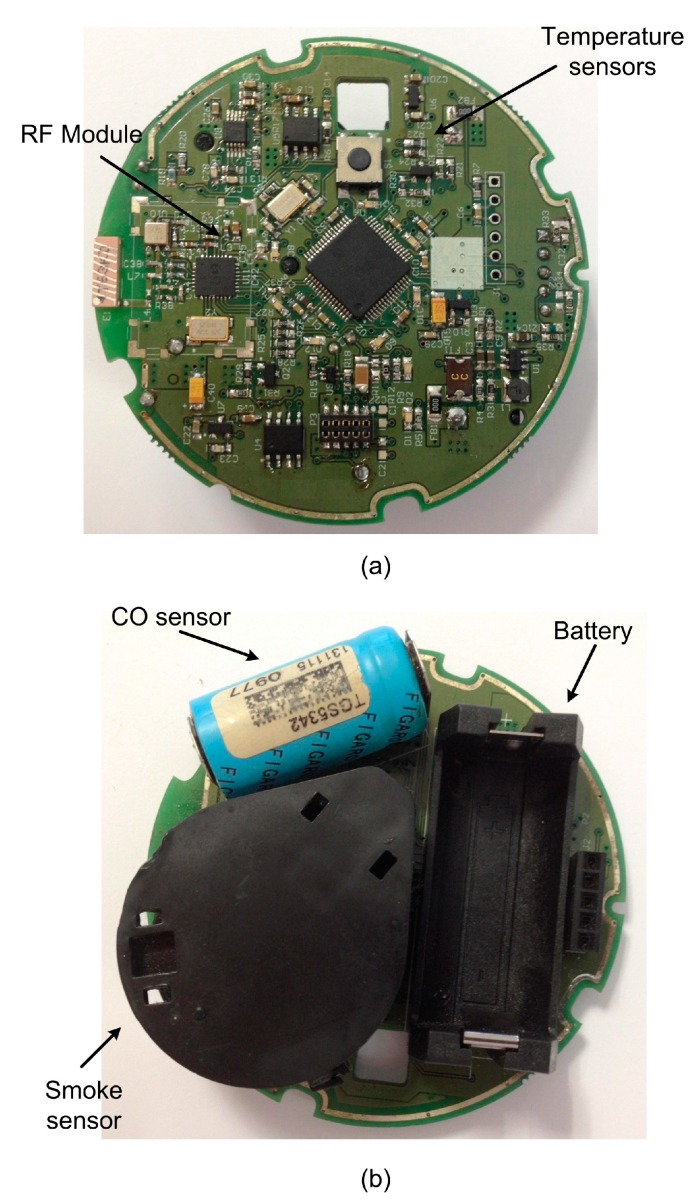
Main board of the fire sensing device. (**a**) Top; (**b**) Bottom.

Figure 5 shows a detailed image of the main board of the developed device. Temperature sensors, the microcontroller, LEDs and the radio module are on the top of the board, whereas the smoke sensor, the carbon monoxide (CO) sensor and the battery are on the bottom.

**Figure 6:**
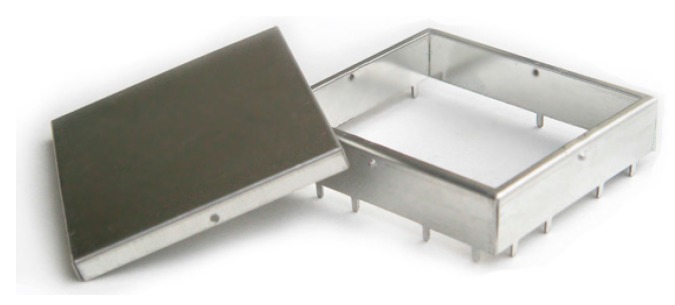
Electromagnetic shield for radio module.

Figure 6 shows the designed electromagnetic shield to avoid interference problems between the RF circuit and adjacent circuits.

There are very few commercial smoke sensors or fire detectors that include a CO sensor. The device developed includes this element and presents a compact solution. In the authors’ opinion, this is the smallest smoke sensor that also includes a CO sensor.

**Figure 7:**
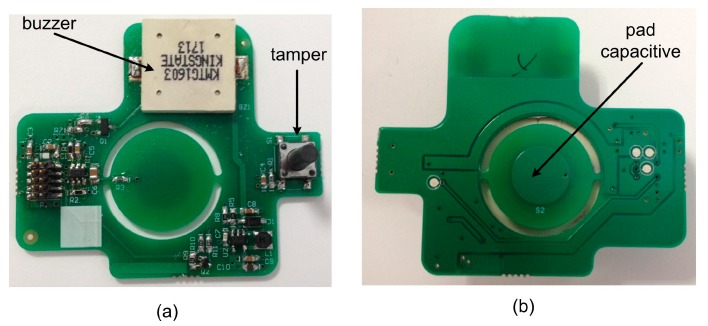
Secondary board. (**a**) Top; (**b**) Bottom.

Concerning the secondary board, its functionality is to capture pulsation events on the capacitive touch button on the top panel of the sensor, and to manage the use of different buzzer sounds to warn the user of smoke/fire presence. This board consists of the buzzer, the capacitive touch pad, the tamper sensor and the circuitry to control those elements as shown in Figure 7. The tamper sensor detects opening of the case or attempted sabotage.

**Figure 8:**
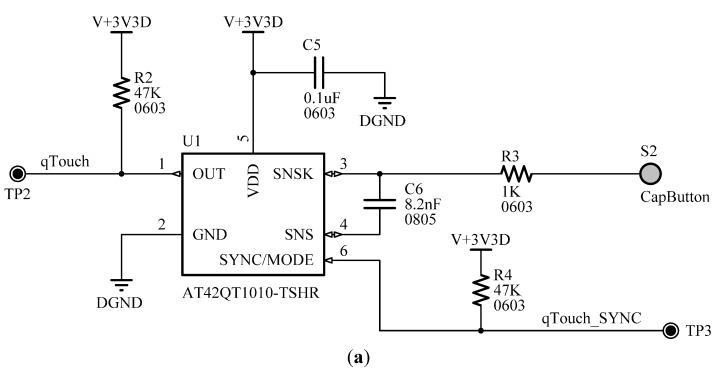

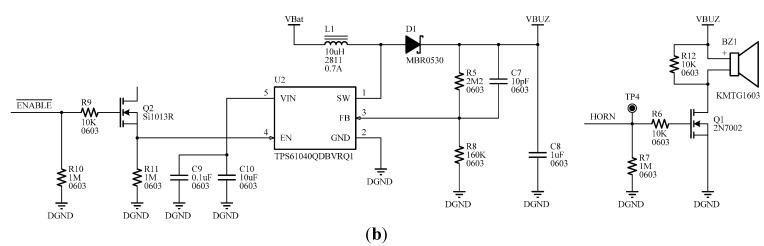
(**a**) Scheme of capacitive sensor for the touch pad; (**b**) Schematic circuit for the buzzer.

The touch button allows the user to turn on or off the wireless fire sensing device. The single-key AT42QT1010-TSHR sensor from Atmel (San Jose, CA, USA) was chosen for touch detection, for its ability to generate an interrupt on touch. This results in a significant improvement in power consumption and autonomy specifications, since the microcontroller does not need to continuously check the state of the button; it can be woken from its low-power state by the interrupt generated by the touch sensor. Figure 8 shows the circuitry of the capacitive sensor for the touch pad and the circuit scheme for the buzzer.

### 2.2. Software

At firmware level, a specific protocol stack has been developed for RF communications. Preliminary test performed on the MiWi protocol proposed by the manufacturer provided unstable results in terms of high latencies and sometimes blocked part of the hardware requiring immediate attention of the processor (MiWi used intensively busy wait cycles). Moreover, encryption algorithms have been implemented to improve the safety of RF communications.

The developed protocol stack uses a SPI controller handled by interrupts to minimize the latency and the busy wait cycles of the microcontroller. The controller for the transceiver is ready to work in the background, so it does not waste busy wait cycles so there is no latency.

The designed stack was made in layers so that each layer could be used independently of the others, allowing the decoupling of the different levels of functionality, which results in greater code portability, better adaptation to new hardware and easier debugging and testing. The same addressing scheme proposed by MiWi has been kept and support a cluster tree network topology (provided by the LLC layer). The design has been done considering minimizing latency and processor usage, hardly busy wait cycles. Therefore, the entire design is based on interrupts for the SPI controller. All functions are implemented as state machines where each state is triggered by an interrupt when the previous operation ends. The communication of asynchronous events such as the reception of new messages or network outage is done thanks to a set of callbacks added to APIs of each layer. Thanks to this, although the development has been somewhat more complex than in the case of stacks as used by MiWi, it has reduced to a minimum the processor usage, and the protocol stack hardly impacts on the other components of the device. The power consumption has been also reduced since the CPU is more in free time.

The sensing node also includes a bootloader functionality. The objective is twofold: first, it allows updating the firmware remotely saving maintenance costs. Secondly, we can verify the proper hardware operation performing a test application. Then, we can finish the test phase and programming loading the final application. At the software level, the bootloader has required to define memory structures, and communication protocols and data sharing. At hardware level, an external flash memory has been included to store the new firmware to load, preventing it from being lost, or that the microcontroller memory may be corrupt due to a power failure or a failure of communications during the remote update process.

### 2.3. Package Design

**Figure 9:**
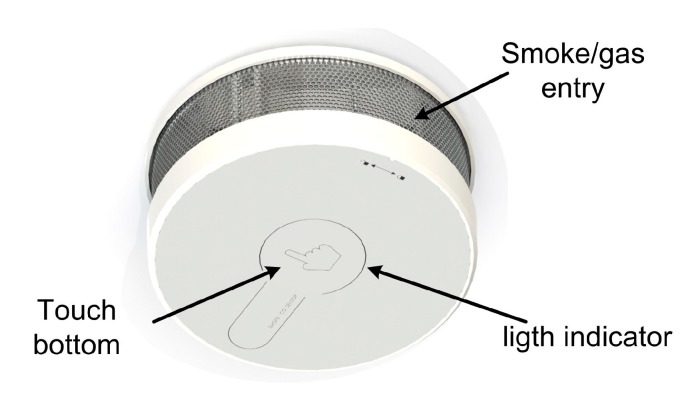
Package design.

**Figure 10:**
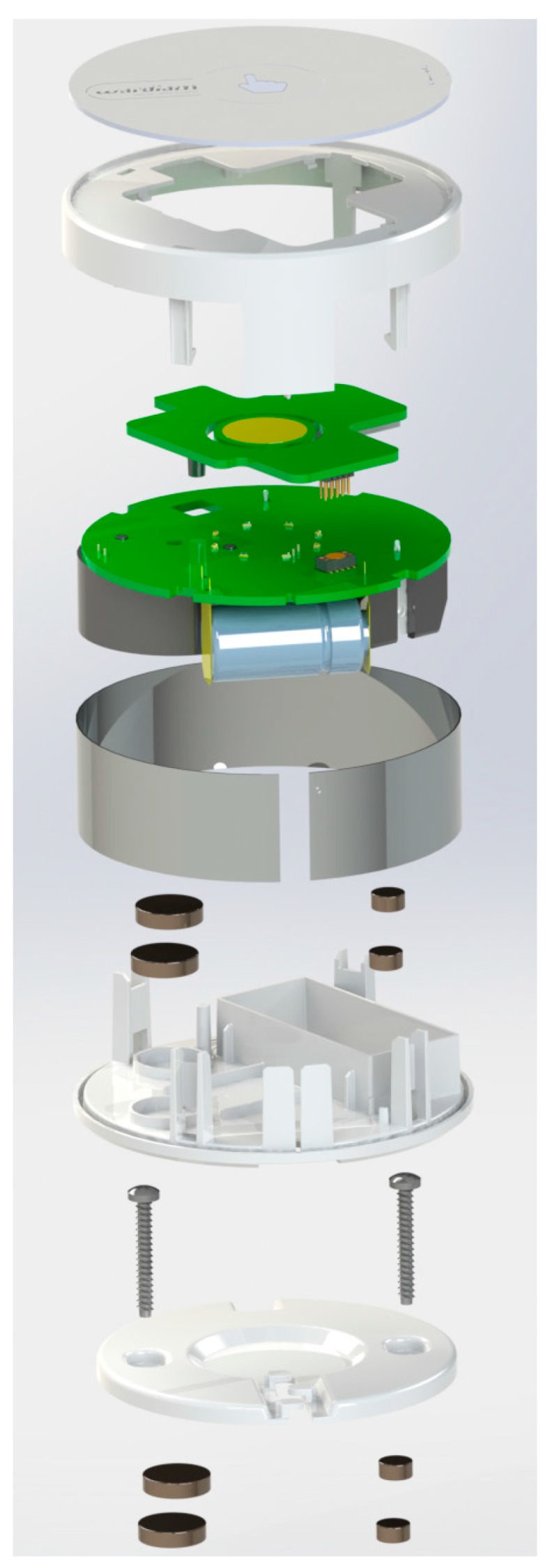
Detail of the fabrication of the entire wireless smoke system.

Figure 9 and Figure 10 show the package designed to protect the sensing device from the environment. This is a cylindrical box with the metal side perforated to allow the entrance of smokes and gases, and a front panel provided with a capacitive touch button. The package does not affect the response time, accuracy of the sensors and the transmission of data. The final dimensions of the sensor package are 70 mm in diameter with a height of 30 mm.

## 3. Results

The proposed hardware and software solutions were validated and the functional performance features of the devices such as, range, flexibility and robustness were assessed. The fire detection device combines three types of sensors: smoke, temperature and carbon monoxide, thus decreasing the number of false alarms due to water vapor, snuff smoke, *etc*.

The fire detection process is performed directly upon the appearance of smoke, allowing locating the site of fire and the fire propagation with accuracy. The use of a CO sensor and temperature sensors increases the reliability of the system and reduces the false alarms.

**Figure 11:**
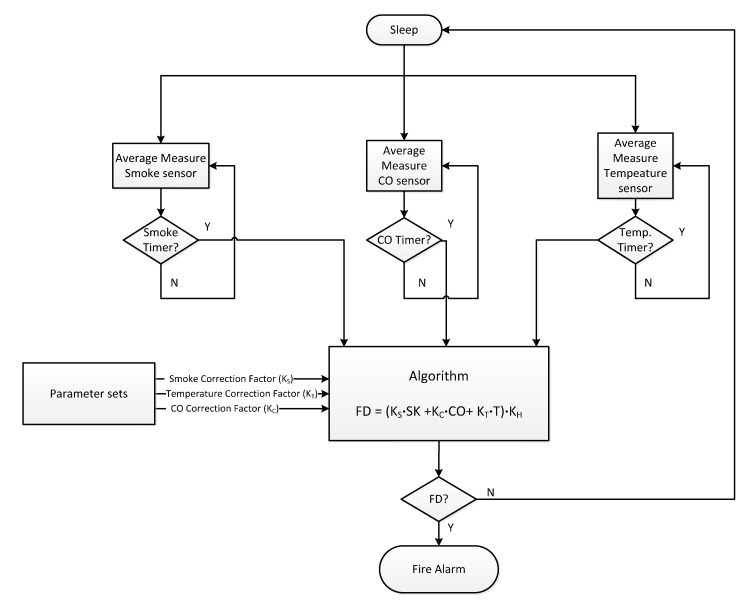
Flow chart of the detection process.

Regarding the alarm detection algorithm, it relies on the joint detection of the three parameters. The detector constantly monitors the three input variables and makes a real-time selection of a suitable linear combination with the form given by *FD* = (*K_S_* *×* *ΔSK* + *K_C_* *×* *ΔCO* + *K_T_* *×* *ΔT*) *×* *K_H_* where *Ks* is the smoke correction factor, *K_C_* is the CO correction factor, *K_T_* is the temperature correction factor, *K_H_* is the installation height, *ΔSK* is the delta in the smoke sensor level, *ΔCO* is the delta in the CO sensor level, and *ΔT* is the delta in the temperature sensor level. Thus, correction factors are assigned to each sensor according to the place of installation in the home achieving a more or less equalized sensitivity. The resulting quantity *FD* represents the instantaneous measure of danger originating from particular fire. By a further suitable weighting of *FD*, different sensitivities for different applications can be achieved with the same detector. The three correction factors *K_S_*, *K_C_* and *K_T_* are assigned to low, medium and high class depending on the place of installation as summarized in Table 1. The correction factors have a degree of membership in each class ranging from 0 to 1. Figure 11 shows the detailed flow chart of the detection process.

**Figure 12:**
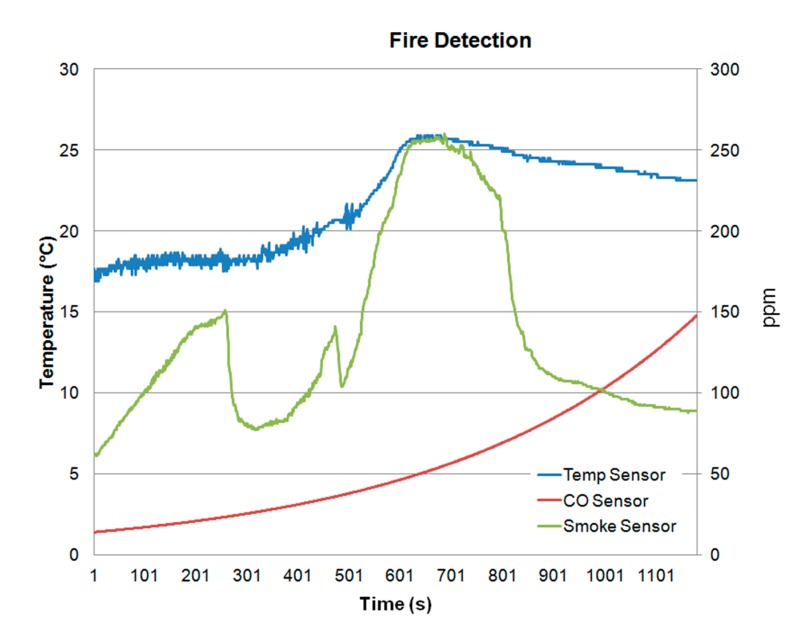
Fire detection.

**Figure 13:**
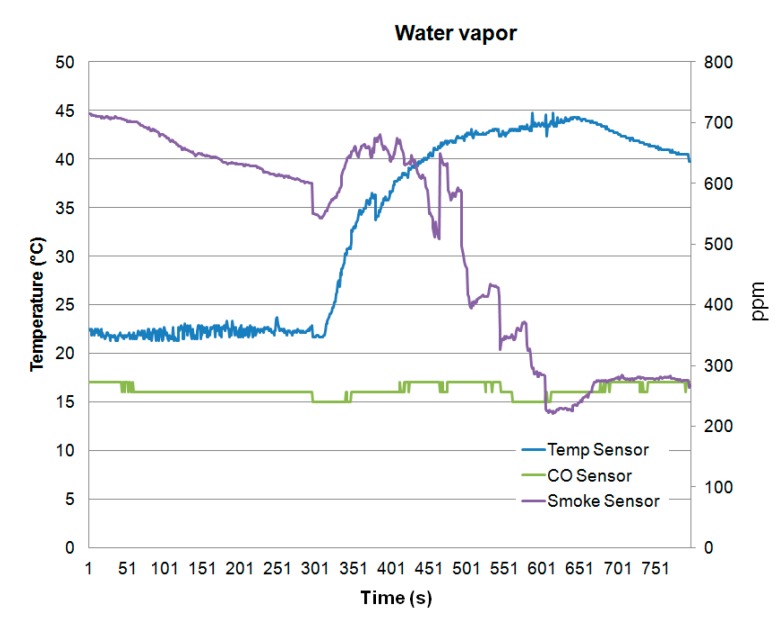
Water vapor detection.

Figure 12 and Figure 13 show different fire detection scenarios. Figure 12 shows the measured values of the three sensors (temperature, smoke and CO) of the wireless fire alarm device. Note that all sensors increase their values. What really discriminates fire detection from other type of vapors (like water vapor) is the presence of CO, which is released during combustion. Thus, Figure 12 shows fire detection and the system triggers an alarm. On the other hand, Figure 13 shows as the developed device avoids a false fire detection since the variations of the sensors detect water vapor due to that the CO concentration is not increased.

**Table 1:** Correction factor against installation.

Room Installation	Smoke (K_S_)	Temperature (K_T_)	CO (K_C_)
Kitchen	Low	Low	Low
Hall	High	High	High
Diving Room	Mid	Low	High
Bedrooms	Mid	High	High
Children Room	Mid	High	High
Car park	Low	High	Low

Several tests were performed in order to verify that the location of the battery and the CO sensor do not obstruct the entry of smoke into the chamber, so that the sensitivity of the detection is not affected by the smoke flow direction. Firstly, the CO sensor capsule was removed; and secondly, both the CO sensor capsule and the battery were removed. The measurement results showed that the same smoke level was reached in all cases, but the response time is faster when the CO sensor and battery are removed. The response time ratio is about 50 samples (around 2.5 s). In most cases this delay is negligible.

The sensor is only powered during data collection. In the sleep mode the transceiver is disabled, but the developed protocol avoids the loss of messages. An estimated power consumption is necessary to guarantee autonomy. The sensing node is always in sleep mode or low power consumption, and only sends a node alive message daily (to notify the system that the sensor is operating). This represents a duty cycle of one second every 24 hours, *i.e.*, a duty cycle of 0.0011%. In terms of consumption, represents an average consumption 16 μAh. Moreover, the sensing device gathers a temperature and smoke measurement every 30 s, which takes an amount of about 50 μs, the average consumption for the acquisition is about 12 μAh. Since the average consumption of the device in sleep mode is approximately 8 μA, the total average current consumption of 36 μAh. For the used battery 6 3V@1600 mAh (CR123 type), the estimated autonomy is 4.75 years.

**Figure 14:**
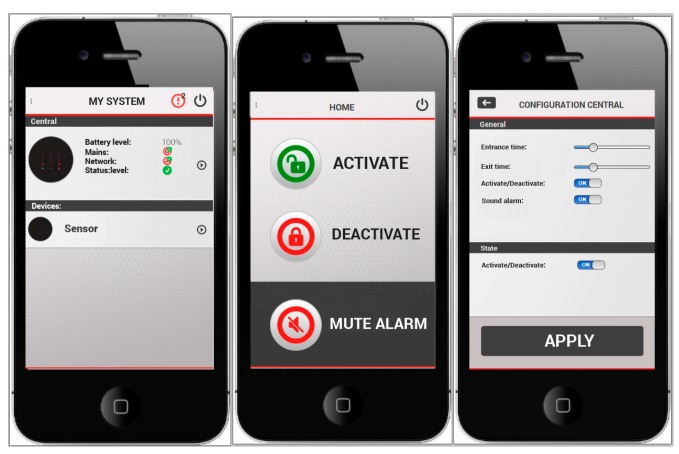
Mobile application.

The designed system can be controlled by means of a mobile application, developed for iOS and Android. It allows the activation and deactivation of the system, and includes additional functionalities. The mobile application has been developed using Objective C and Java for iOS and Android, respectively. It is connected to application servers and notification services both Apple and Google to generate push notifications for alarms, and that the user can receive instant information about any event occurring on the system. Figure 14 shows the mobile application with different functionalities.

## 4. Conclusions

A low cost low power wireless fire alarm device is presented. The sensing device uses the combination of a smoke sensor, temperature sensors and a CO sensor to provide better fire alarm detection. The device reduces false-alarm rates and provides high reliability. The compact design and the wireless approach ensure a system installation that does less damage to buildings, convenient to place nodes and easy maintenance. Hardware components have been chosen to assure functionality and low power consumption. The power management at software level allows the system to save energy, as the device only consumes 36 μAh ensuring an autonomy of almost 5 years with the installed battery. A reliable and robust packaging has also been designed to protect the sensing device.

## References

[1] Ahrens M. (2011). Smoke Alarm Presence and Performance in U.S. Home Fires. Fire Technol..

[2] Feo-Arenis S., Westphal B., Dietsch D., Muñiz M., Siyar Andisha A. (2014). The Wireless fire alarm system: Ensuring conformance to industrial standards through formal verification. Lecture Notes in Computer Science.

[3] Aspey R.A., Brazier K.J., Spencer J.W. (2005). Multiwavelength sensing of smoke using a polychromatic LED: Mie extinction characterization using HLS analysis. IEEE Sens. J..

[4] Li J., Wang S., Dou Z., Yang Z. (2001). Discrimination of smoke particles using infrared photoelectrical detection. Int. J. Infrared Millim. Waves.

[5] Müller K., Loepfe M., Wieser D. (2006). Optical simulation for fire detectors. Fire Safety J..

[6] Shi M., Bermak A., Chandrasekaran S., Amira A., Brahim-Belhouari S. (2008). A committee machine gas identification system based on dynamically reconfigurable FPGA. IEEE Sens. J..

[7] Sawada A., Higashino T., Oyabu T., Takei Y., Nanto H., Toko K. (2008). Gas sensor characteristics for smoldering fire caused by a cigarette smoke. Sens. Actuators B.

[8] Skinner A.J., Lambert M.F. (2006). Using smart sensor strings for continuous monitoring of temperature stratification in large water bodies. IEEE Sensors J..

[9] Chen S.-J., Hovde D.C., Peterson K.A., Marshall A.W. (2007). Fire detection using smoke and gas sensors. Fire Safety J..

[10] Aleksic Z.J. (2004). The analysis of the transmission-type optical smoke detector threshold sensitivity to the high rate temperature variations. IEEE Trans. Instrum. Meas..

[11] Cheon J., Lee J., Lee I., Chae Y., Yoo Y., Han G. (2009). A single-chip CMOS smoke and temperature sensor for an intelligent fire detector. IEEE Sens. J..

[12] Chen Z., Shi Z., Guo Q. (2013). Design of wireless sensor network node for carbon monoxide monitoring. Telecommun. Syst..

[13] Zeng Y., Sreenan C.J., Sitanayah L., Xiong N., Park I.H., Zheng G. (2011). An emergency-adaptive routing scheme for wireless sensor networks for building fire hazard monitoring. Sensors.

[14] Rawat P., Singh K.D., Chaouchi H., Bonnin J.M. (2014). Wireless sensor networks: A survey on recent developments and potential synergies. J. Supercomput..

[15] Rault T., Bouabdallah A., Challal Y. (2014). Energy efficiency in wireless sensor networks: A top-down survey. Comput. Netw..

[16] Lloret J., Garcia M., Bri D., Sendra S. (2009). A wireless sensor network deployment for rural and forest fire detection and verification. Sensors.

[17] Baker B.C. (1994). Photodiode Monitoring with Op Amps.

[18] Maxim Integrated Products (2002). Amplifier Provides Signal Conditioning for Piezofilm Sensors.

[19] Yamashita M. (2002). Amplification Circuit for Electric Charge Type Sensor. U.S. Patent Appl..

